# Well-differentiated fetal adenocarcinoma of the lung: positron emission tomography features and diagnostic difficulties in frozen section analysis—a case report

**DOI:** 10.1186/s40792-020-00910-0

**Published:** 2020-06-29

**Authors:** Shuhei Hakiri, Takayuki Fukui, Hideki Tsubouchi, Ayako Sakakibara, Shingo Iwano, Toyofumi F. Chen-Yoshikawa

**Affiliations:** 1 Department of Thoracic Surgery, Nagoya University Graduate School of Medicine, 65 Tsurumai-cho, Showa-ku, Nagoya, 466-8550 Japan; 2 Department of Pathology and Laboratory Medicine, Nagoya University Hospital, Nagoya, Japan; 3 Department of Radiology, Nagoya University Graduate School of Medicine, Nagoya, Japan

**Keywords:** Well-differentiated fetal adenocarcinoma, Fluorodeoxyglucose positron emission tomography, Frozen section

## Abstract

**Background:**

Well-differentiated fetal adenocarcinoma (WDFA) of the lung is a rare disease that resembles fetal lung tubules. Most of previous reports concerning WDFA have focused on histological features, while there are few reports describing radiological features. In addition, there are no reports evaluating the difficulty of intraoperative diagnosis of WDFA with frozen section. We report a case of WDFA and review the radiological features of WDFA including the findings of F-18 fluorodeoxyglucose positron emission tomography (FDG-PET) and assess the difficulty of intraoperative diagnosis with frozen section.

**Case presentation:**

A chest radiography performed in a 20-year-old female revealed a mass in the hilum of the right lung. Computed tomography revealed a well-defined mass measuring 3.5 × 3.0 cm in diameter in the right upper lobe, whereas PET showed a high accumulation of FDG. The most likely diagnosis was clinical T2aN0M0 stage 1B non-small cell lung cancer. A right S^3^ segmentectomy was performed via thoracotomy, and a benign tumor that was possibly an adenoma was intraoperatively diagnosed based on frozen section analysis. The mass was a solid tumor measuring 2.9 × 2.5 cm in diameter. Microscopically, the tumor comprised abundant glands with single or double layers of nonciliated cells and bronchial structures resembling a fetal lung. Rounded morules of polygonal cells were frequently observed. Immunohistochemistry revealed that nuclei and cytoplasm of the tumor cell were positive for β-catenin. Finally, the postoperative pathological diagnosis was well-differentiated fetal adenocarcinoma of the lung, and completion right upper lobectomy and mediastinal lymph node dissection were conducted 1 month after the initial segmentectomy. No residual tumor or lymph node metastasis was identified, and the final pathological stage was pT1cN0M0 stage 1A3. The patient did not wish to receive any adjuvant therapy. At the 1-year follow-up, no evidence of recurrence was noted.

**Conclusions:**

Here, we report a rare case of well-differentiated fetal adenocarcinoma of the lung that was difficult to diagnose based on radiological evaluations including FDG-PET and intraoperative diagnosis using frozen section analysis.

## Background

Fetal adenocarcinoma was first described by Kradin et al. in 1982 as a subtype of pulmonary blastoma [1]; thereafter, Kodama et al. introduced the term “fetal adenocarcinoma” in 1984 [2]. In 1998, Nakatani et al. established two case types of fetal adenocarcinoma of the lung: low-grade and high-grade. The low-grade type is known as well-differentiated fetal adenocarcinoma (WDFA) of the lung, predominantly occurring in young females with a good prognosis [3]. Conversely, the high-grade type is poorly differentiated, predominantly occurring among elderly males with a poorer prognosis than the low-grade type. WDFA is a rare disease, accounting for only 0.1% of all pulmonary neoplasms. It consists of tubules of glycogen-rich, nonciliated cells that resemble fetal lung tubules, and most previous reports concerning WDFA have focused on histological features. Conversely, reports describing the radiological features of this disease are limited [4, 5]. To the best of our knowledge, only one case has presented the findings of F-18 fluorodeoxyglucose positron emission tomography (FDG-PET) [6]. In addition, no reports evaluating the difficulty of establishing an intraoperative diagnosis of WDFA using frozen section analysis are available. Here, we report a case of WDFA and review the radiological features of this disease, including FDG-PET findings, and assess the difficulty of establishing an intraoperative diagnosis using frozen section analysis.

## Case presentation

**Fig. 1:**
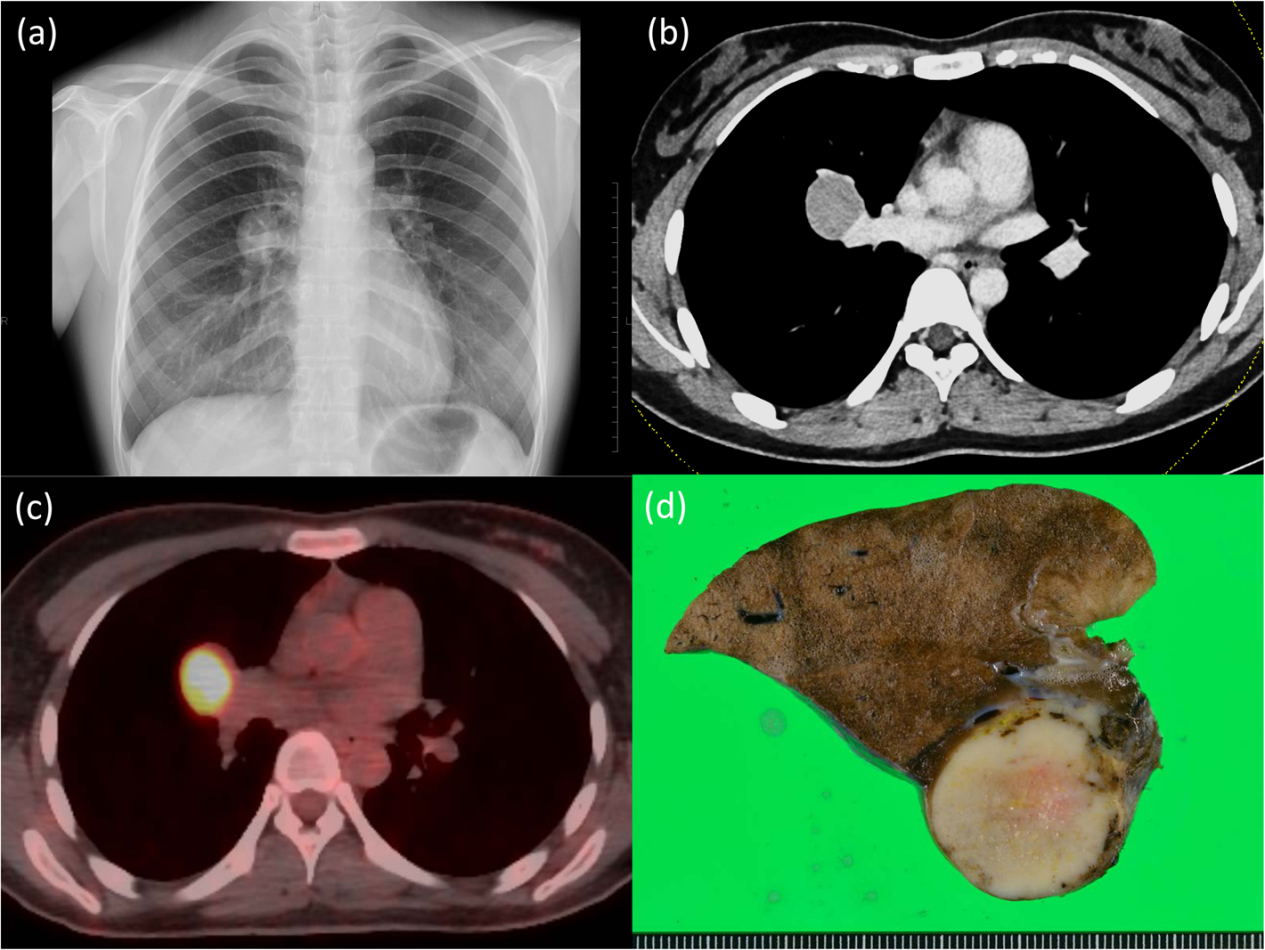
Radiological findings and macroscopic appearance of the tumor. **a** Chest X-ray revealed a shadow in the right middle lung field. **b** Computed tomography revealed a tumor in the right upper lobe. **c** F-18 fluorodeoxyglucose positron emission tomography revealed high accumulation of FDG in the lesion of the right upper lobe. **d** Macroscopic appearance of the tumor, which appeared as a solitary, white-colored, well-defined, and solid component

A 20-year-old woman with no smoking history presented a round-shaped shadow in the right middle lung field on a chest X-ray during her medical checkup on employment (Fig. 1a), while her chest X-ray 2 years ago had no abnormalities. She exhibited no symptoms and had no comorbidities. Contrast-enhanced computed tomography (CT) of the chest showed a lobulated solid mass in the hilum of the right lung measuring 3.5 × 3.0 cm in diameter, which was homogenously enhanced in the early phase scan without any intratumoral fat or calcification identified (Fig. 1b). FDG-PET revealed a high accumulation of FDG in the mass wherein the maximum standard uptake values were 7.1 and 8.4 in the early and delayed phases, respectively (Fig. 1c). The levels of all serum tumor markers of lung cancer such as carcinoembryonic antigen, cytokeratin 19 fragment, and pro-gastrin-releasing peptide were within normal ranges. Based on the radiological findings, the mass was suspected to be either an invasive lung cancer, a lymphoma, Castleman’s disease, a solitary fibrous tumor, or an inflammatory pseudotumor. A transbronchial lung biopsy was performed, but the specimen was too small for histological diagnosis. It was decided that the most likely diagnosis of the mass was clinical T2aN0M0 stage 1B non-small cell lung cancer as per Union for International Cancer Control (UICC) 8th edition criteria. Accordingly, the patient underwent anterolateral thoracotomy through the fifth intercostal space. Intraprocedurally, it was detected that the mass arose from the S^3^ of the right upper lobe and that its surface facing the interlobular space was covered with visceral pleura. The mass had not invaded the middle lobe, but it was close to the hilar vessels. Thus, wedge resection with the mass was considered to be difficult, and right S^3^ segmentectomy was performed. Intraoperative frozen section analysis revealed that the tumor contained branched gland ducts along with columnar epithelial cells with poor nuclear dysplasia arranged in papillary growth patterns. Therefore, the tumor was diagnosed as a benign pulmonary lesion (e.g., papilloma or adenoma), and the operation was completed without deploying any additional procedures.

**Fig. 2:**
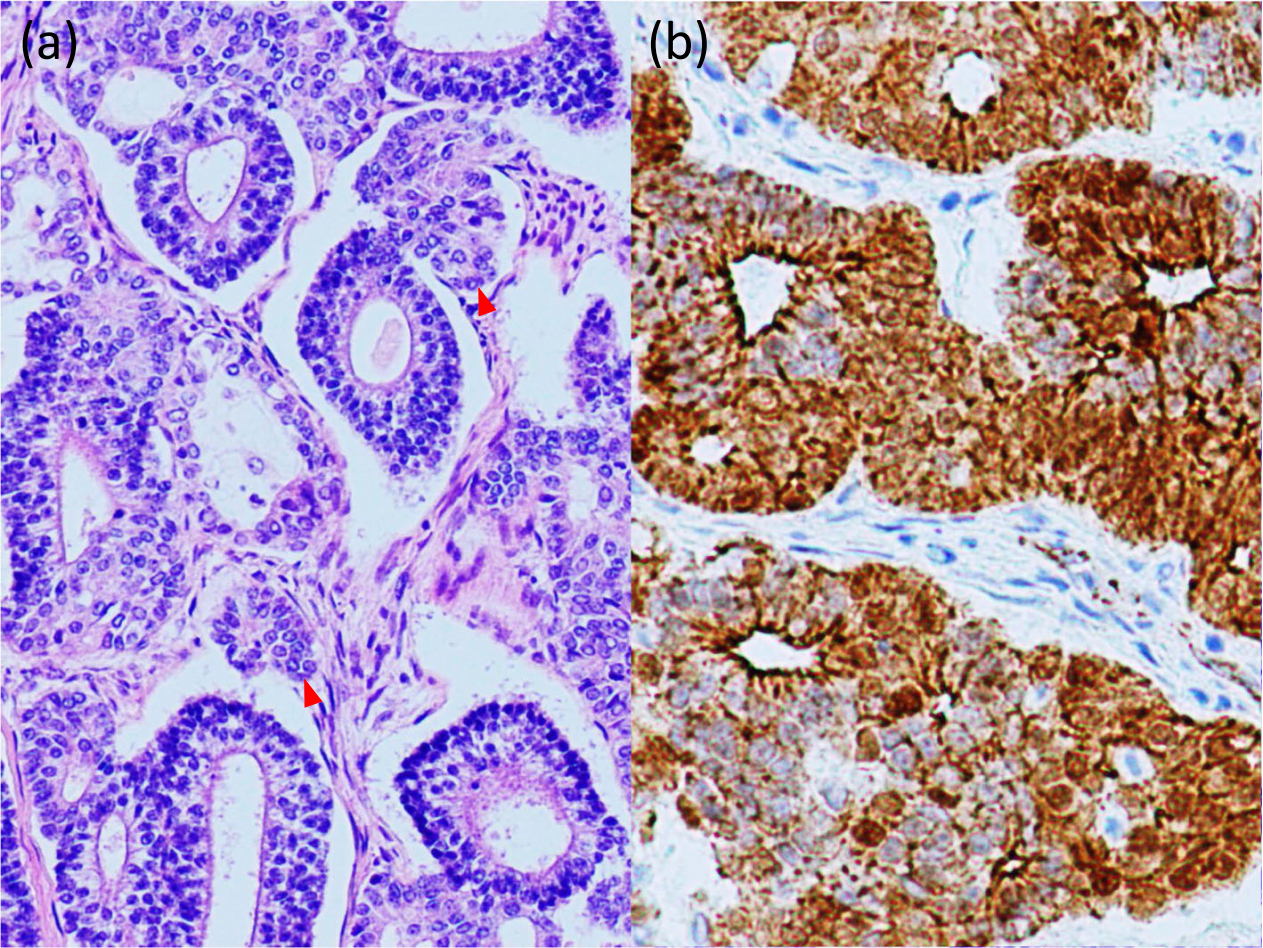
Microscopic examination of the surgical resection specimen. **a** Hematoxylin and eosin-stained section showed glands comprising single or double layers of nonciliated cells and some morule formations (red arrowhead). **b** Immunohistochemical examination showed a positive result for β-catenin

Macroscopically, the mass was a solitary, white-colored, well-defined, and solid component measuring 2.9 × 2.5 cm in diameter (Fig. 1d). These findings were seldom found in conjunction with necrotic or hematomatous lesions. Microscopically, it consisted of abundant glands with single or double layers of nonciliated cells and bronchial structures that resembled the tubular epithelium of a fetal lung. Rounded morules of polygonal cells with eosinophilic cytoplasm were frequently observed (Fig. 2a). Immunohistochemistry showed that nuclei and cytoplasm of the tumor cells were positive for β-catenin (Fig. 2b) and negative for p53, nuclei of the tumor cells were positive for thyroid transcription factor 1, and the cytoplasm was positive for synaptophysin. Based on the abovementioned morphological patterns and immunohistochemical profile, the tumor was finally diagnosed as a WDFA.

At 1 month after the initial operation, the patient underwent completion right upper lobectomy and systematic lymph node dissection. Anterolateral thoracotomy was performed via the fifth intercostal space, same with the incision in the initial operation; further, the lower lobe and chest wall surrounding the wound were widely attached, and both the major and minor fissures were firmly adhered. Although peeling off the adhesions was difficult, the remaining upper lobe was successfully removed without any complications such as vessel injury, lung damage, and massive bleeding. According to the pathological findings, no residual tumor or lymph node metastasis was present, and the final pathological stage was pT1cN0M0 stage 1A3 (UICC, 8th edition). The postoperative course was uneventful, and the patient was discharged on postoperative day 6. She did not wish to receive any adjuvant therapy. At 1-year after the second operation, the patient was well without any evidence of recurrence.

## Discussion

In the present report, we observed two important clinical issues. First, WDFA showed a high accumulation of FDG on PET. Second, the intraoperative diagnosis of WDFA appeared difficult using histological examination with frozen section analysis.

Force et al. reported the first case of WDFA with FDG-PET findings [7]. Their case showed a high accumulation of FDG, similar to that observed in our patient. Fetal lung, as opposed to the adult lung, is dependent on glucose metabolism, and fetal lung tissue expresses glucose transporter isoform 1 (GLUT1) [8]. GLUT1 has been reported in most cases of lung carcinoma, particularly those of squamous cell carcinoma; furthermore, in adenocarcinoma, low rates of cell differentiation and larger tumor sizes have been correlated with the expression of GLUT1. WDFA demonstrated a substantial uptake of FDG regardless of the nature of tumor malignancy. It may be difficult to differentiate WDFA from other lung cancers using FDG-PET because of high FDG accumulations on both WDFA and other lung cancers.

In addition, WDFA showed well-defined oval or lobulated solid lesions on contrast-enhanced CT, which are often homogenously enhanced without calcifications. Yamakawa et al. reported that intratumoral enhancing vasculature was noted in the early phase of dynamic CT, whereas a persistent and plateau enhancement was noted in the delayed phase [9]. However, these findings were not specific and similar to those of lung cancer, inflammatory pseudotumor, solitary fibrous tumor, lymphoma, sarcoidosis, and Castleman’s disease. Therefore, the preoperative diagnosis of WDFA appears difficult based on radiological findings only.

Furthermore, to the best of our knowledge, no case of WDFA has been diagnosed using intraoperative frozen section analysis, whereas some cases have been diagnosed via aspiration cytology preoperatively. Geisinger et al. reported four cases of WDFA diagnosed using aspiration cytology with immunochemical staining; however, these authors concluded that the cytological diagnosis of WDFA may be challenging [10]. Finally, they proposed that immunochemical studies using markers of epithelial and neuroendocrine differentiation and using β-catenin will definitely support the diagnosis of WDFA where warranted. Moreover, Proctor et al. suggested that β-catenin immunohistochemistry, as part of a panel of immunostaining, would aid in this difficult diagnosis in the setting of a limited biopsy, such as fine-needle aspiration biopsy [11]. Notable histological features of WDFA include substantial morule formation morphologically and positive β-catenin staining during nuclear and cytoplasmic immunohistochemistry; these findings provide important evidences for achieving an accurate diagnosis. It may be difficult to intraoperatively diagnose WDFA via frozen section analysis if only a small specimen is examined and immunostaining is not performed.

Momozane et al. summarized 31 cases of WDFA resected in Japan, and they asserted the difficulty of establishing a definitive diagnosis preoperatively [12]. Among these 31 cases, 10 were histologically diagnosed with malignant tumor preoperatively; nine of these cases were lung cancers, such as adenocarcinoma, squamous cell carcinoma, and small cell carcinoma, whereas the remaining case was diagnosed as pulmonary blastoma including WDFA. A pathologist with little experience may have difficulty confirming WDFA because of the rarity of the condition. However, if patients are young females of approximately 30 years of age showing a high accumulation of FDG on a well-defined solid lung nodule, WDFA should be listed as one of the differential diseases and the necessity of anatomical lung resection and lymph node dissection should be considered. Local recurrence of WDFA following limited resection and lymph node metastasis has been reported [13]. Adjuvant chemotherapy and radiation are unlikely to provide substantial benefit and are thus not recommended [14].

## Conclusions

We found that WDFA is associated with a high accumulation of FDG on PET scan. Moreover, WDFA may be difficult to diagnose using only small biopsy specimens and using frozen section analysis intraoperatively. Thoracic surgeons and pathologists alike should consider WDFA as one of the differential diagnoses when relatively young women demonstrate a well-defined solid lung lesion with a high accumulation of FDG. If there is strong possibility of WDFA, the necessity of anatomical resection should be considered.

## Data Availability

Not applicable.

## Abbreviations

WDFA: Well-differentiated fetal adenocarcinoma
FDG-PET: Fluorodeoxyglucose positron emission tomography
CT: Computed tomography
UICC: Union for International Cancer Control
GLUT1: Glucose transporter isoform 1
